# Supplementary material for: Alcohol use among emergency medicine department patients in Tanzania: A comparative analysis of injury versus non-injury patients
Source: PLOS Glob Public Health. 2023 Nov 1;3(11):e0001900. doi: 10.1371/journal.pgph.0001900 (PMC10619788; doi:10.1371/journal.pgph.0001900)
Supplement: Attachment — Submitted filename: Response to Reviewers - inj vs non.docx [file pgph.0001900.s006.docx]

August 22nd, 2023

To Whom It May Concern:

We appreciate the helpful suggestions presented to us by our reviewers and thank them for the opportunity to improve our manuscript. We have incorporated the changes requested by all reviewers in our attached submission, with our specific modifications outlined below. Our revised manuscript is attached with this resubmission packet.

Reviewer #1:

**Comment**: The authors present an interesting study evaluating differences in the rates of alcohol use and alcohol-related behaviors between IPs and NIPs to guide future EMD interventions. While I understand and accept their findings, my concern is that their statistical analysis remains incomplete. It would have been great to see some statistical tests or hypothesis testing (chi-square, t-test) evaluating the significance of factors influencing the differences in the rates of alcohol usage and behaviors between the two groups. I believe their study would be of great benefit in guiding future interventions, but it requires further analysis to be able to lend weight to their recommendations. I invite them to resubmit this paper after performing a more in-depth analysis of the data they have described.

*Response: We thank the reviewer for this suggestion. We have performed a more in-depth analysis as recommended. This resubmission contains Wilcoxon rank sum testing, Pearson's Chi-squared testing, and Fisher's exact testing as appropriate for all variables presented in this manuscript. We have also added a description of our updated analysis procedure under “Methods: Analysis.”*

Reviewer #2:

**Comment**: The introduction requires substantial improvement. State the rationale of the study carefully and clearly.

*Response: We thank the reviewer for this feedback. We have made substantial changes to our introduction section as well as added our rationale for this study as suggested. While a full description of our changes can be seen within the revised introduction, in brief, injury patients with alcohol use disorder within KCMC’s Emergency Medicine Department (EMD) were the initial patient population for locally planned alcohol-reduction interventions. However, it is important to know if this planned intervention population is the most appropriate option or if there is a need to expand intervention delivery to a larger population. Given the already established infrastructure for the intervention, the rationale of this study is thus to determine the populations most at-risk for alcohol use disorder within KCMC’s EMD and assess if other EMD populations could benefit from an intervention.*

**Comment**: Please include the conceptual framework of your study.

*Response: We thank the reviewer for this suggestion and have included our conceptual framework in our section “Methods: Study Design.” This addition reads, ‘The SBIRT program matrix stands as the guiding conceptual framework for this study given the aim of better-informing program implementation and effectiveness.’*

**Comment**: Can you please explain what is AUDIT score and how it is important to include in the study?

*Response: We thank the reviewer for these clarifying questions and appreciate the opportunity to strengthen our manuscript by answering them. AUDIT scores serve as a screening tool for measuring alcohol consumption and alcohol-related problems. The tool itself incorporates metrics for measuring the quantity, frequency, and consequences of alcohol use which make it more sensitive in a multicultural, challenging setting like ours to quantify the content of alcohol consumed by patients. The use of AUDIT is important in this study as a positive score triggers the initiation of further clinical care and our SBIRT protocol. We have included this information under the section “Methods: Variables” to better explain AUDIT and its importance within this study.*

**Comment**: Can you perform regression analysis?

*Response: We appreciate this suggestion from the reviewer. However, as our study is centered on understanding differences between injury and non-injury patient groups, we feel that performing a regression analysis would fall outside of our intended objective. Further, given our study sample size, we believe that it would be out of scope to look at factors that influence injury or non-injury status. We have instead included further testing, including Wilcoxon rank sum, Pearson's Chi-squared, and Fisher's exact testing in this revision to better explore the statistical significance between our variables. If, after reading this, there remains a specific association that the reviewer would like us to analyze, please let us know, and we will do our best to accommodate it.*

**Comment**: Please use either one or two decimal point?

*Response: We thank the reviewer for this suggestion and have now reported all values to two decimal points.*

**Comment**: The table are missing captions.

*Response: We thank the reviewer for bringing this to our attention. We have added captions to all tables presented in this manuscript.*

**Comment**: There are so many grammatical mistakes and spelling mistakes. please correct them. Also recheck the references?

*Response: We appreciate this feedback from the reviewer. We have performed a thorough grammar and references review and have corrected all errors identified.*

**Comment**: Can you please include policy recommendation.

*Response: We thank the reviewer for this suggestion. We have included a policy recommendation in our discussion section.*

**Comment**: Usman, M., Anand, E., Akhtar, S. N., Umenthala, S. R., Anwar, T., & Unisa, S. (2022). Prevalence and correlates of alcohol and tobacco consumption among research scholars: evidence from a cross-sectional survey of three Indian universities. Drugs, Habits and Social Policy, (ahead-of-print).

*Response: We thank the reviewer for bringing this paper to our attention. We have added it as a reference within our discussion section in reference to the correlation between mental wellness and substance use. This reference helps us demonstrate that this link is found in other settings.*

Reviewer #3:

The authors performed a reanalysis of a previously run study that took place over several months in the emergency room of a single hospital in Tanzania. The authors previously had seen that trauma patients or (injured patients IP) had high rates of alcohol use. They wanted to know about the epidemiology of alcohol use in the non-injured (NIP) patient population. They also compared usage between IP and NIPs.

**Comment**: Patients were excluded if they were unable to consent. It is possible (perhaps even probable) that the rate of alcohol use is different between patients who are able to consent and those who are unable to (even after a waiting period). This exclusion makes sense for the study but should be acknowledged in the limitations.

*Response: We thank the reviewer for this suggestion. An acknowledgment of this has been added to our strengths and limitations section and reads, ‘...patients who remained unable to consent over a 24-hour period were excluded from study participation. While this was deemed necessary for ethical and safety reasons, it may have impacted study demographics given potentially different alcohol use patterns in this patient group than those who were able to consent.’*

**Comment**: It appears that females were oversampled in this study. The reasons given for this are sound but it will affect the reported overall incidence of alcohol use in both IP and NIPs (likely resulting in an underestimate). This should also be acknowledged.

*Response: We thank the reviewer for bringing this to our attention. We have incorporated this point into our limitations section, which reads, ‘The purposeful oversampling of female patients in the study from which this data originates impacted our reported incidence of alcohol use when looking at amalgamated IP and NIP statistics in Table 2, resulting in a likely underestimation of true intake. To account for this bias, we have also reported statistics separated by gender, as seen in Table 3.’*

**Comment:** I recommend using a consistent number of decimal points in the tables (probably 1)

*Response: We thank the reviewer for this suggestion and have now reported all values to two decimal points.*

**Comment**: The authors spend a lot of time in their results examining the alcohol use differences between IP and NIPs. I understand that this was the stated purpose of the paper. However, it appears that the proposed intervention is the use of alcohol intervention programs. It is mildly interesting to know the relative use of alcohol between IP and NIPs. What is more of a take-home message is that alcohol use is high in both groups and, therefore, interventions can be more broadly applied. The authors do highlight this appropriately in the discussion.

*Response: We thank the reviewer for this comment and much appreciate this reflection.*

**Comment**: I do not see any tests of significance between groups in the tables. Was this considered?

*Response: We thank the reviewer for raising this question. Significance testing had originally been considered and is now included in this resubmission. Wilcoxon rank sum testing, Pearson's Chi-squared testing, and Fisher's exact testing were performed as appropriate for all variables presented in this manuscript. We have also added a description of our updated analysis procedure under “Methods: Analysis.”*
